# Vacuolization in embryos on days 3 and 4 of *in vitro* development: Association with stimulation protocols, embryo development, chromosomal status, pregnancy and neonatal outcomes

**DOI:** 10.3389/fendo.2022.985741

**Published:** 2022-10-19

**Authors:** Longbin Chen, Shuoping Zhang, Yifan Gu, Yangqin Peng, Zenghui Huang, Fei Gong, Ge Lin

**Affiliations:** ^1^ Institute of Reproductive and Stem Cells, School of Basic Medicine, Central South University, Changsha, China; ^2^ Research Department of CITIC Xiangya Reproductive and Genetic Hospital, Changsha, China; ^3^ Key Laboratory of Reproductive and Stem Cell Engineering, National Health and Family Planning Commission, Changsha, China

**Keywords:** embryo vacuoles, euploidy, clinical outcomes, neonatal outcomes, self-correction

## Abstract

**Study question:**

Is vacuolization in embryos on Days 3 and 4 associated with parent-related factors, stimulation protocols, embryo development, embryo ploidy, pregnancy and neonatal outcomes?

**Study design, size, duration:**

This is a retrospective cohort study that comprised 5,703 embryos from 611 patients who underwent preimplantation genetic testing and time-lapse monitoring of their embryos from August 2017 to September 2021.

**Main results:**

Embryo vacuolization on Days 3 and 4 is associated with the LH level on the day of the hCG trigger and the number of retrieved oocytes. Compared to vacuole-negative embryos, the rates of blastocyst formation and good-blastocyst formation was significantly lower in vacuole-positive embryos. We observed no significant difference in the rates of euploidy, implantation, ongoing pregnancy, and live birth between vacuole-positive and vacuole-negative embryos. In vacuole-positive embryos, the embryos of which the vacuole-positive blastomeres were involved in embryo compaction exhibited significantly higher mosaicism rate compared with those of which the vacuole-positive blastomeres were not involved in embryo compaction.

**Conclusion:**

Vacuolization in embryos on Days 3 and 4 is associated with reduced blastocyst formation rate and high-quality blastocyst rate. Blastocysts had a low mosaicism rate if the vacuole-containing cells were rejected in compaction process, which supports the hypothesis that exclusion of abnormal blastomeres from compaction is a self-correction mechanism.

## Introduction

Embryo vacuole is a cytoplasmic inclusion which contains liquids from perivitelline space (1), and emerges from the oocyte to blastocyst stages (2). Researchers have speculated that vacuoles originate from abnormal endocytosis during PB1 expulsion, micromanipulation needle rupture of cell membrane during intracytoplasmic sperm injection (ICSI), or fusion of small vesicles secreted by organelles (1, 2). Although scientists have assessed the effect of vacuoles in human oocyte and showed that the presence of vacuoles in oocyte was a marker of aging and associated with low pregnancy rate (1, 3, 4), studies on vacuoles in human embryo are limited. Ebner et al. found that the later the vacuoles appeared, the more serious the effect observed on blastocyst formation (2). Therefore, more attention needs to be afforded on the clinical outcomes of embryos with vacuoles present on Day 3 and Day 4 of embryo development.

Zhang et al. focused on vacuolization on Days 3 and 4 by time-lapse imaging, and found it was negatively correlated with embryo development (5). But they lack data about chromosome status and live birth outcomes of vacuole-positive embryos. Mayer’s showed that there was no live birth when all embryos in one cycle were affected by vacuoles present on Day 3 and Day 4, speculating that vacuoles may be the manifestation of apoptosis or necrosis (6). Van Blerkom et al. speculated that large vacuoles may hinder the movement of the spindle into the polar position, resulting in chromosome separation error (1). However, a case report visualizing the spindle revealed that large vacuoles are not associated with spindle displacement (7). So it is uncertain whether vacuoles can affect the process of mitosis and result in embryo mosaicism.

Lagalla et al. observed the phenomenon that cells were excluded in the process of embryo compaction, they found the excluded cells had a high aneuploid rate, which suggested the exclusion of aneuploid cells during embryo compaction was a mechanism of embryo self-correction (8). Since embryo compaction reflects a process of rapid and violent cell movement, it is difficult to tracking and locate the excluded cells. Vacuoles (as an obvious cellular feature) can assist embryologists in better tracking the locations of cells and thereby allow them to obtain more accurate data, which benefits for exploring the correlation between cell exclusion and embryo development.

In this study, we investigated the association between embryo vacuolization on Days 3 and 4 and parent-related factors, ovarian-stimulation regimens, embryo development, embryo ploidy, clinical and neonatal outcomes.

## Materials and methods

### Patients

A total of 611 patients whose embryos underwent preimplantation genetic testing (PGT) and time-lapse embryo monitoring from August 2017 to September 2021 at the Reproductive and Genetic Hospital of CITIC-Xiangya were included in this study. The Ethics Committee of CITIC-Xiangya approved our study.

### Ovarian stimulation, ICSI and embryo culture

Ovarian-stimulation protocols were principally executed according to the subject’s ovarian reserve (9). 5,000–10,000 IU hCG (Pregnyl, Merck, NJ, United States) was subcutaneous injected when two-thirds of the follicles reach 18 mm. Oocytes were collected 34–36 hours after hCG administration by transvaginal ultrasonography, and were placed in fertilization medium (Vitrolife, Goteborg, Sweden) at 37°C in an atmosphere of 6% CO_2_ for 3–4 hours prior to ICSI.

Fertilization was assessed 16–18 hours after ICSI, and only the normally fertilized zygotes were placed individually into the microwell of a specifically designed center-well culture dish (Vitrolife, Budapest, Sweden) and cultured in sequential media (Vitrolife, Goteborg, Sweden) to the blastocyst stage. During this entire period, the embryos remained in a 37°C incubator with 6% CO_2_ and 5% O_2_ and were monitored with a Primo Vision time-lapse system (Vitrolife).

### Time-lapse imaging and data collection

**Figure 1:**
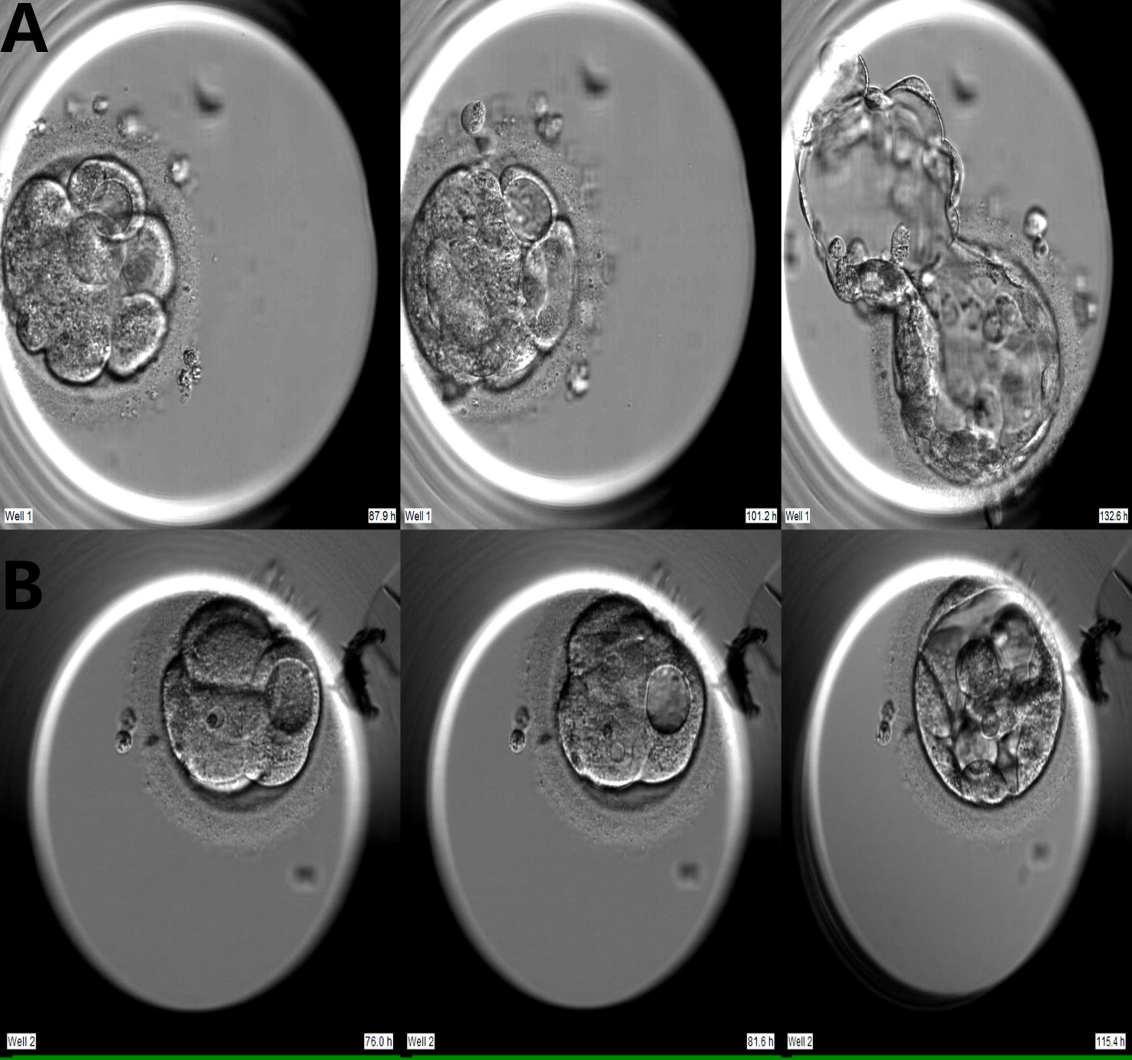
Two developmental models of vacuole-containing cells during embryo compaction. **(A)** Vacuole-containing cells that did not participate in compaction; **(B)** Vacuole-containing cells that participated in compaction.

Embryo development from zygote to blastocyst was retrospectively analyzed using computer-equipped software (Primo Vision Analyzer, Vitrolife). The vacuole is a nearly circular membrane-bound cytoplasmic inclusion with strong refractive properties (10). The vacuole-positive embryo were defined as embryo that first appears vacuole on Day 3 and Day 4. The vacuole-positive cycles had at least one embryo with vacuoles on Day 3 or Day 4. When embryos showed vacuoles on Days 3 and 4, we counted the total number of vacuoles, the proportion of cells affected by vacuoles (the number of vacuole-containing cells divided by the total number of cells in embryo), and we measured the largest vacuole in each embryo. We tracked the developmental destiny of vacuole-containing cells (**Figure 1**).

### Blastocyst grading

Blastocyst grading was based upon the criteria proposed by Gardner and Schoolcraft (11, 12), and included three morphological parameters: the degree of blastocyst expansion, the number and arrangement of inner cell mass (ICM) and trophectoderm (TE). Blastocysts which score were ≥ 4BC or 4CB (i.e., the degree of blastocyst expansion = 4, 5, or 6; ICM and TE ratings were not C at the same time) reached the standard of biopsy, and those whose score were ≥ 4BB (i.e, the degree of blastocyst expansion = 4, 5, or 6; ICM grade = A or B; and TE grade = A or B) were considered to be of high quality (11, 13).

### Blastocyst biopsy, cryopreservation, genetic testing and embryo transfer

On the morning of Day 5, an opening of about 25 µm in the zona pellucida of the embryo was made by a Zilos TK laser (Hamilton Thorne, USA). Between Day 5 and Day 7, the hatching or hatched blastocysts were biopsied. Approximately 3–8 trophectoderm cells were aspirated with a biopsy needle and separated by laser-mediated drilling. Blastocysts were vitrified 1–2 hours after biopsy using a Kitazato Vitrification Kit (Kitazato Biopharma, Shizuoka, Japan) in combination with closed High Security Vitrification Straws (CryoBio System, Lucerne, Switzerland). We implemented chromosomal analysis using the GenomePlex Single Cell Whole Genome Amplification Kit (Sigma-Aldrich, MO, USA) and next-generation sequencing. Embryos were defined as euploid if no alteration to the reference base line was observed, and embryos were defined as mosaic if the equivalent of 30–70% of cells were abnormal. Less than 30% of abnormal cells were classified as euploid, and over 70% of abnormal cells were classified as aneuploid.

Single blastocyst transfer was performed in all cycles, the euploid blastocyst with the highest morphological score was selected (14). The warmed blastocyst was placed in blastocyst medium (G2.5, Vitrolife) for 2–6 hours. The re-expanded blastocysts were considered as survival and suitable for transfer. Implantation was defined as gestational sac can be seen by ultrasonography after 4 weeks of pregnancy. Ongoing pregnancy was defined as the presence of a gestational sac with fetal heartbeat at week 12 after embryo transfer. Low birth weight was defined as less than 2500 g. Preterm birth was defined as birth before 37 weeks of gestation.

### Statistical analysis

Continuous data were presented as mean ± SD and compared using Student’s t-test for normally distributed data. Categorical data are presented as percentages, and the χ^2^-test and Fisher’s exact test were used for comparisons. Statistical analysis was carried out using Statistical Package for Social Sciences (SPSS version 25.0, Chicago, IL, USA). We initially employed univariate regression analysis to screen the risk factors related to vacuole-positive cycle, and then exploited multivariate regression analysis to obtain the ultimate independent risk factors. The proportion of vacuolar cells, the sizes of vacuoles, and the number of vacuoles were grouped according to the interquartile spacing. The linear-by linear association test was used to examine linear associations between the proportion of vacuolated cells, the sizes of vacuoles, or the number of vacuoles and high-quality blastocyst rate. A two-sided *P* value of < 0.05 was considered statistically significant.

## Results

A total of 611 cycles from 611 patients were included. Of the 611 cycles, there were 321 vacuole-positive cycles and 290 vacuole-negative cycles. Among 5,703 embryos in the 611 cycles, 796 embryos showed vacuoles on Day 3 and Day 4, with an incidence of 13.95%.

### Baseline and stimulation data

**Table 1** indicated that female age, BMI, basic endocrine level, male age, semen source, semen quality and ovarian-stimulation protocols had no difference between the vacuole-positive and vacuole-negative cycles. The LH level on the day of the hCG trigger and the number of retrieved oocytes were significantly lower in vacuole-positive cycles. The multivariable analysis retained both the LH level (odds ratio (OR) = 0.900 (95% CI: 0.810–0.999), *P* = 0.047) and the number of retrieved oocytes (OR = 1.030 (95% CI: 1.002 – 1.060), *P* = 0.035) in the final model (**Supplementary Table 1**).

**Table 1:** Demographic and stimulation data of vacuole-positive and vacuole-negative cycles.

	Vacuole-positive cycles (321)	Vacuole-negative cycles (290)	*P*
Female			
Female age (years)	34.40 ± 5.45	34.47 ± 5.39	0.878
Female body mass index (kg/m²)	21.74 ± 2.21	21.70 ± 2.33	0.809
AMH (ng/mL)	5.20 ± 3.50	5.46 ± 4.57	0.433
No. of antral follicles	20.17 ± 9.64	21.77 ± 13.55	0.09
Basal OGTT (mmol/ml)	5.25 ± 0.32	5.23 ± 0.27	0.183
Basal IRT (mmol/ml)	9.33 ± 4.72	9.14 ± 2.72	0.548
IRT resistance index	2.00 ± 0.44	2.02 ± 0.38	0.722
Basal FSH (mIU/ml)	5.85 ± 1.66	5.70 ± 1.91	0.286
Basal LH (mIU/ml)	3.84 ± 2.04	3.88 ± 2.85	0.836
Basal E2 (pg/mL)	67.44 ± 281.36	46.46 ± 137.56	0.25
Basal PRL (mg/mL)	17.60 ± 32.15	16.15 ± 12.27	0.473
Basal P (nmol/ml)	0.68 ± 2.16	0.48 ± 1.95	0.243
Basal T (ng/mL)	0.67 ± 2.80	0.59 ± 2.76	0.745
FT3 (pg/mL)	2.80 ± 0.57	2.81 ± 0.66	0.856
FT4 (pg/mL)	2.22 ± 8.45	1.63 ± 2.86	0.255
BS (mmol/ml)	5.12 ± 0.79	5.06 ± 0.90	0.453
TSH (mol/l)	1.98 ± 1.05	1.95 ± 1.05	0.726
Ovarian stimulation			
Ovarian stimulation protocol			0.062^a^
GnRH-agonist protocol	176	133	
GnRH-antagonist protocol	68	81	
Others	77	76	
Total stimulatory dose (IU)	2570.99 ± 1544.93	2501.72 ± 1529.29	0.578
E2 level on the trigger day (pg/mL)	3164.67 ± 1316.29	3069.96 ± 1576.98	0.419
LH level on the trigger day (pg/mL)	2.02 ± 1.33	2.30 ± 1.78	0.023
P level on the trigger day (pg/mL)	0.93 ± 0.44	0.96 ± 0.65	0.463
No. of retrieved oocytes	13.35 ± 6.35	14.47 ± 5.29	0.019
Proportion of immature oocytes (%)	16.81 ± 13.98	17.2 ± 26.95	0.279
Male			
Male age (years)	39.82 ± 2.39	39.52 ± 3.54	0.974
Semen source			0.337^a^
Fresh semen	290	266	
Frozen semen	19	12	
Percutaneous epididymal or testicular sperm aspiration	3	6	
Sperm vitality (%)	21.74 ± 2.21	21.70 ± 2.33	0.809
Sperm count (ml)	0.96 ± 0.18	0.96 ± .19	0.694

OGTT, oral glucose tolerance test; IRT, insulin release test; FT3, serum-free triiodothyronine; FT4, serum-free tetraiodothyronine, TSH, thyroid-stimulating hormone; BS, blood sugar; Sperm vitality was the proportion of live, membrane-intact spermatozoa as determined by either dye exclusion or osmoregulatory capacity under hypoosmotic conditions.

a P values for testing overall differences between the groups.

### Embryological and clinical outcomes of vacuole-positive and -negative embryos

We noted no difference in the incidences of mosaicism and euploidy between vacuole-positive and vacuole-negative embryos. The vacuole-positive embryos exhibited significantly reduced blastocyst formation rate (50.6% *vs.* 54.8%) and high-quality blastocyst formation rate (46.7% *vs.* 53.4%) (**Table 2**). Implantation, ongoing pregnancy and live birth rates did not differ between vacuole-positive and vacuole-negative embryos. The neonatal outcomes of birthweight, low birthweight rate, birth length, preterm birth rate and birth defect rate were comparable between the two groups.

**Table 2:** Embryological, clinical, and neonatal outcomes for vacuole-positive and vacuole-negative embryos.

	Vacuole-positive embryos	Vacuole-negative embryos	*P*
Blastocyst formation (%)	403/796 (50.6)	2691/4907 (54.8)	0.027
High-quality embryo rate (%)	168/360 (46.7)	1438/2691 (53.4)	0.018
Mosaicism (%)	13/245 (5.3)	71/1703 (4.2)	0.413
Euploidy (%)	123/232 (53)	860/1632 (52.7)	0.927
Implantation rate (%)	35/63 (55.6)	311/472 (65.9)	0.107
Ongoing pregnancy rate (%)	27/63 (42.9)	251/472 (53.2)	0.124
Live-birth rate (%)			
total	26/63 (41.3)	217/472 (46)	0.481
Monozygotic twins	2	5	/
Mode of delivery			*0.422^a^*
natural delivery (%)	7/63 (28)	46/472 (21)	
cesarean (%)	18/63 (72)	173/472 (79)	
Birth weight			
singleton	3254.35 ± 368.01	3338.52 ± 566.15	*0.168*
Monozygotic twins	1775 ± 694.62	2525 ± 599.79	*0.076*
Low birth weight			
singleton (%)	1/25 (4.0)	12/230 (5.2)	*0.793*
Monozygotic twins	1	1	/
Birth length (cm)			
singleton	50.19 ± 1.33	50.02 ± 1.33	*0.81*
Monozygotic twins	/	/	/
Preterm birth			
singleton (%)	2/26 (7.7)	24/217 (11.1)	*0.6*
Monozygotic twins	0	1	/
Birth defect			
singleton (%)	2/34 (5.9)	14/222 (6.3)	*0.527*
Monozygotic twins	0	0	/

a P values for testing overall differences between the groups.

The relationship between different vacuole types and blastocyst quality is shown in **Table 3**. With the increasing proportion of cells affected by vacuoles, the rate of high-quality blastocyst significantly decreased from 28.6% to 13.7% (P trend = 0.000). With the increasing size of vacuoles, the rate of high-quality embryos significantly decreased from 29.5% to 15.1% (P trend = 0.000). When the number of vacuoles increases from 1~2 to 11~30, the high-quality blastocyst rate dropped from 26.0% to 20.4%, with no significant difference among four groups (P = 0.573, P trend = 0.25).

**Table 3:** Relationship between different vacuole types and blastocyst quality.

Type of vacuole	Interquartile				P	P for trend
Proportion of vacuolar cells (%)	6.25~16.7	16.8~30	30.1~41.7	41.8~100		
High-quality blastocyst rate (%)	59/206 (28.6)a	59/209 (28.2)a	28/162 (17.3)a,b	26/190 (13.7)b	0.000	0.000
Maximum vacuole size (μm)	5~24	25~31	32~38	39~86		
High-quality blastocyst rate (%)	62/210 (29.5)a	48/186 (25.8)a,b	36/199 (18.1)b	26/172 (15.1)b	0.002	0.000
Number of vacuoles	1~2	3~5	6~10	11~30		
High-quality blastocyst rate (%)	50/192 (26.0)a	49/231 (21.2)a	50/231 (21.6)a	23/113 (20.4)a	0.573	0.25

a, b: Values with different superscripts are significantly different (P < 0.05).

### Embryological and clinical outcomes of partially compacted *vs*. fully compacted vacuole-positive embryos

Of the 796 vacuole-positive embryos, 600 reached the compaction stage (75.4%). Of these, vacuole-containing cells were involved in compaction in 120 embryos (VAC+C), and vacuole-containing cells were not involved or extruded in the compaction process in 480 embryos (VAC-C) (**Figure 1**). In addition, 2,930 vacuole-negative embryos developed to the compaction stage (NC).

The blastocyst formation rate in NC group (83.3%) was significantly higher than both the VAC-C (65.2%) and VAC+C groups (69.2%). The mosaicism, euploidy, implantation, ongoing pregnancy and live-birth rates of VAC-C group were similar to those of NC group. In contrast, the implantation, ongoing pregnancy and live-birth rates of the VAC+C group were significantly lower than those of NC group. There were no significant differences in rates of blastocyst formation, high-quality blastocyst, implantation, ongoing pregnancy and live birth between VAC+C and VAC-C groups. VAC+C group showed a significantly higher mosaicism rate than VAC-C and NC groups. Perinatal outcomes, including delivery mode, birth weight, low birth weight, birth length, preterm birth and birth defect, were not different among the three groups (**Table 4**).

**Table 4:** Embryological and clinical outcomes in VAC+C, VAC-C, and NC groups.

	VAC-C=1	VAC+C =2	NC=0	*P* *1 vs. 2*	*P* *1 vs. 0*	*P* *2 vs. 0*
Blastocyst formation rate (%)	313/480 (65.2)	83/120 (69.2)	2440/2930 (83.3)	0.413	0.000	0.000
High-quality Blastocyst rate (%)	130/249 (52.2)	38/88 (43.2)	1306/2440 (53.5)	0.145	0.739	0.056
Mosaicism (%)	7/187 (3.7)	6/55 (10.9)	61/2292 (2.7)	0.038	0.351	0.004
Euploidy (%)	94/180 (52.2)	27/49 (55.1)	773/1478 (52.3)	0.72	1.0	0.772
Implantation rate (%)	29/48 (60.4)	6/15 (40.0)	282/412 (68.4)	0.165	0.259	0.027
Ongoing pregnancy rate (%)	23/48 (47.9)	4/15 (26.7)	228/412 (55.3)	0.147	0.360	0.035
Live-birth rate (%)	23/48 (47.9)	3/15 (20)	198/412 (48.1)	0.055	1.0	0.037
Mode of delivery				0.250^a^	0.283^a^	0.363^a^
natural delivery (%)	7/23 (30.43)	0/3 (0)	43/198 (21.71)			
cesarean (%)	15/23 (65.22)	3/3 (100)	155/198 (78.28)			
Birth weight	3058.33 ± 713.94	2850 ± 150	3330.32 ± 549.96	0.287	0.784	0.987
Low birth weight (%)	3/23 (13.04)	0/3 (0)	11/198 (5.6)	0.506	0.167	0.675
Birth length (cm)	50.46 ± 1.27	49 ± 1	50.04 ± 1.32	0.235	0.967	0.586
Preterm birth (%)	2/23 (8.70)	0/3 (0)	21/198 (10.61)	0.595	0.776	0.551
Birth defect (%)	1/23 (4.35)	0/3 (0)	7/198 (3.53)	0.713	0.804	0.741

a P values for testing overall differences between the groups.

## Discussion

In this study we investigated the association between cytoplasmic vacuoles on Day 3 and Day 4 embryos and the corresponding patient-related clinical factors. We uncovered a link between the number of oocytes retrieved, LH level on the day of trigger and embryo vacuolization. LH is essential for normal folliculogenesis and oocyte maturation in the natural ovulatory menstrual cycle. Insufficient endogenous LH secretion may lead to defective luteal function, and bears implications for the asynchronous maturation of cytoplasm and the nucleus (15). Two studies found that low levels of LH on the day of triggering were associated with low oocyte maturation rate (16, 17). Further studies are required to discern the underlying explanation for our results.

Thomas et al. claimed that vacuoles disturbed the movement of the MII spindles and organelles in the oocyte and thus affected cell division (18). Although it is generally accepted that vacuoles are deleterious to embryo development (2, 5), it remains unclear whether vacuoles exert longer-term effects on clinical pregnancy and live birth. In this study, we observed that vacuolization impaired the competence of blastocyst formation and blastocyst quality, but if blastocysts from vacuole-positive embryos were transferred, their pregnancy and neonatal outcomes were unaffected, which suggests the vacuole-associated variation in preimplantation embryo has no long-term impacts on embryo development and health of offspring.

Zhang et al. found that the more the number of blastomeres affected by vacuoles, the worse the embryo’s development outcome (5), which was similar to our results. We further confirmed the linear relationship between the proportion of vacuolated cells and the rate of high-quality blastocysts. We also recorded the size and number of vacuoles. It turned out there was a significant negative correlation between vacuole size and blastocyst quality. Although the rate of high-quality blastocysts also decreased with the increase of the number of vacuoles, there was no statistical difference. Our results suggest that the proportion of cells affected by vacuoles and the vacuole size are correlated with low embryo quality, and should be taken into account when selecting the embryo for transfer.

It has been reported that aneuploidy and mosaicism rates were lower in blastocysts than in cleavage-stage embryos (19–22). One study suggested that aneuploid embryos exhibited a tendency for gradual normalization of chromosomal status from the cleavage-embryo to blastocyst stage (19). Mertzanidou et al. further showed that the process of self-correction started at the morula stage (23), and Lagalla et al. speculated that excluding or extruding abnormal cells during peri-compaction period may be a mechanism subserving embryo self-correction (8). In our study, 80% of embryos with vacuoles underwent partial compaction. The mosaicism rate of VAC-C embryos was significantly lower than that of VAC+C embryos. These results were consistent with a previous hypothesis of embryo self-correction where abnormal blastomeres do not participate in embryo compaction. We further compared the embryo development and clinical outcomes of NC embryos with VAC-C and VAC+C embryos, and showed that the mosaicism rate for VAC+C embryos was the highest among the three groups. It is worth noting that the implantation rate, ongoing pregnancy rate and live birth rate of the NC group were significantly higher than the rates for the VAC+C group, but similar to those of the VAC-C group. Even when euploid embryos were transferred, the involvement of vacuole-positive blastomeres in compaction might introduce other abnormalities that affected embryo development.

According to our results, vacuole-positive embryos showed similar pregnancy and neonatal outcomes to vacuole-negative embryos, thus the vacuole-positive embryos could be considered by clinicians to be an option for embryo transfer. Vacuole-positive blastomeres that participate in embryo compaction could constitute an indicator for embryo selection when embryos with vacuoles were the only choice available.

There were two limitations for the present study. First, the sample size of transferred embryos was limited, which might reduce the statistical power for finding differences between groups. Second, due to the limitations of our technology platform, we can only detect 30% of the mosaicism.

In conclusion, the occurrence of embryo vacuole on Days 3 and 4 was found to be related to the LH level on the day of the hCG trigger and the number of retrieved oocytes. Embryo vacuole exerts effects on blastocyst formation and quality, but not on subsequent embryo development. The exclusion of vacuole-positive blastomeres during embryo compaction may be a mechanism of self-correction that allows the normal continued development.

## Data availability statement

The raw data supporting the conclusions of this article will be made available by the authors, without undue reservation.

## Ethics statement

The studies involving human participants were reviewed and approved by the Ethics Committee of CITIC-Xiangya. The patients/participants provided their written informed consent to participate in this study.

## Author contributions

LC and SZ was in charge of conception and design and drafting the article. YG was in charge of acquisition of data. PY and ZH were in charge of analysis and interpretation of data. FG and GL were in charge of advice on experimental design and revise results. All authors contributed to the article and approved the submitted version.

## Acknowledgments

The authors wish to thank the patients and staff at the Reproductive and Genetic Hospital of CITIC-XIANGYA.

## Conflict of interest

The authors declare that the research was conducted in the absence of any commercial or financial relationships that could be construed as a potential conflict of interest.

## Publisher's note

All claims expressed in this article are solely those of the authors and do not necessarily represent those of their affiliated organizations, or those of the publisher, the editors and the reviewers. Any product that may be evaluated in this article, or claim that may be made by its manufacturer, is not guaranteed or endorsed by the publisher.
